# Cell-SELEX Identifies a “Sticky” RNA Aptamer Sequence

**DOI:** 10.1155/2017/4943072

**Published:** 2017-01-17

**Authors:** Partha Ray, Rebekah R. White

**Affiliations:** ^1^Department of Surgery, UC San Diego School of Medicine, La Jolla, CA, USA; ^2^Department of Surgery, Duke University School of Medicine, Durham, NC, USA

## Abstract

Cell-SELEX is performed to select for cell binding aptamers. We employed an additional selection pressure by using RNAse to remove surface-binding aptamers and select for cell-internalizing aptamers. A common RNA sequence was identified from independent cell-SELEX procedures against two different pancreatic cancer cell lines, indicating a strong selection pressure towards this sequence from the large pool of other available sequences present in the aptamer library. The aptamer is not specific for the pancreatic cancer cell lines, and a similar sequence motif is present in previously published internalizing aptamers. The identified sequence forms a structural motif that binds to a surface protein, which either is highly abundant or has strong affinity for the selected aptamer sequence. Deselecting (removing) this sequence during cell-SELEX may increase the probability of identifying aptamers against cell type-specific targets on the cell surface.

## 1. Introduction

Aptamers are nucleic acid polymers that are selected by Systematic Evolution of Ligands by Exponential enrichment (SELEX). Although initially described to select ligands for small molecules and purified proteins in vitro [1, 2], the process itself has evolved over years and is now regularly used to select ligands that can bind to cells. Cell-SELEX, as the procedure has been termed, uses cells to generate aptamers that bind them with high affinity [3]. Selectivity has been incorporated into the selection scheme by introducing steps such as counterselections, where cycles of positive and negative selections are performed. Positive selections are done against the cell type that is the desired target for aptamer binding. The cells chosen for negative selections are generally a cell type that is similar to the positive cell type yet varies in certain traits that can be targeted. For example, if one desires to generate an aptamer that would target certain cancer cell type, a corresponding normal cell type would be used as the negative selection target. Consequently, the aptamers that binds to common targets that are shared between the positive and negative cells are removed from the selection, whereas aptamers that display specificity for the cancer cell targets are preferentially selected. Using this type of selection scheme, several aptamers have been generated that display high affinity and specificity against their targeted cells [4].

However, one of the major drawbacks of cell-SELEX is that, despite performing positive-negative selection cycles, we often select for aptamers that do not display specificity for the target cells. They either bind to the target and the control cells with equal affinity or display modest preference in binding to the targeted cells over the control cells [5]. Measures such as increasing stringency during the positive selection cycles and using competitor nucleic acids (e.g., transfer-RNA (tRNA) or salmon sperm DNA) to mask nonspecific interactions between the aptamer library and the cell surface have been utilized to improve specificity, albeit with limited dividend [5].

The idea behind performing the positive-negative selection strategy, as mentioned earlier, is to find proteins on the cell surface of the target cell type that are different or absent from the corresponding normal cells. This is challenging since most of the proteins displayed on the cell surface are common to many cell types [6]. We reasoned that the highly abundant targets present on the cell surface are responsible for the nonspecificity of the aptamers that are selected. These common cell surface targets can also have high affinity towards aptamers owing to their charge or structure, contributing to this problem.

We hypothesized that identifying these targets, or the aptamers that bind them, could mitigate the problem. Either these interactions could be blocked or the nonspecific aptamer sequence(s) could be deselected during cell-SELEX to provide a better opportunity to select aptamers that are specific for the desired cell type.

## 2. Materials and Methods

### 2.1. Cell-SELEX

MiaPaCa-2 and Panc-1 cells were plated (~2 × 10^6^ cells/well) and grown in 6-well plates in DMEM + 10% FBS media the day before cell-SELEX, under standard cell growth conditions.

The sequence of the DNA template oligonucleotide, 5′ primer, and the 3′ primer are 5′-TCGGGCGAGTCGTCTG-*n*_40_-CCGCATCGTCCTCCCTA-3′ (*n*_40_ represents a 40-nucleotide random region with equimolar quantities of A, T, C, and G), 5′-GGGGGAATTCTAATACGACTCACTATAGGGAGGACGATGCGG-3′, and 5′-TCGGGCGAGTCGTCTG-3′, respectively. DNA oligonucleotides were synthesized by Oligos etc., Inc.. The double stranded DNA template was prepared by annealing the DNA template oligonucleotide to the 5′ primer and then filling in the template with Exo-Klenow (NEB). The starting RNA library was generated by in vitro transcription using natural purines and 2′-fluoro-modified pyrimidines (TriLink Biotechnologies) and a modified T7 RNA polymerase and then gel-purified.

For round one (R1), the cells were incubated with 4 nmole of Sel III (2′-fluoro-pyrimidine modified aptamer library 5′-TCGGGCGAGTCGTCTG-*n*_40_-CCGCATCGTCCTCCCTA-3′ as described previously [10]) in 1 mL of DMEM (Gibco®) + 10% FBS media, for 1 hour at 37°C (tissue culture incubator) under the same conditions used for growing the cells. After incubation, the media were removed, and cells were stringently washed 5 times (5x) in DPBS buffer containing magnesium and calcium chloride (Gibco). Next, cells were lysed directly on the plates with 1 mL TRIzol® reagent (Thermo Fisher Scientific), and total RNA was extracted per the manufacturers' protocol. Subsequently, total RNA was reverse transcribed [11] with the aptamer specific primer. The reaction mixture was treated with RNAse (RiboShredder™, RS) (Epicentre Biotechnologies) to remove RNA and, after ethanol precipitation of the cDNA, it was PCR amplified to generate DNA templates for transcription of the next round. For round two (R2), in order to increase the stringency of the selection, the ratio of RNA to cells was decreased; 400 pmoles of RNA pool was incubated with 0.5 × 10^6^ cells/well. The cells were incubated with the RNA pool for 1 hr and washed 5x with the same buffer as done previously in R1. However, in order to further increase the stringency of the selection, the cells were treated with protease-trypsin (Gibco) with the intent of removing RNA bound to the cell surface proteins, before the TRIzol extraction of total RNA. In round 3 (R3), the same protocol for R2 was adapted with further increase of stringency by adding RNAse cocktail (RiboShredder, RS) (Epicentre Biotechnologies) after the trypsin treatment in order to select for only cell-internalized RNA [11, 12]. From round four (R4) through round seven (R7), the protocol for R3 was repeated with the exception of decreasing the RNA–cell incubation time, from 1 hour to 30 minutes with the intent of further increasing the stringency of the selection pressure.

### 2.2. High Throughput Sequencing (HTS) Data and Analysis

For HTS, the DNA templates obtained after each round (R1 through R7) were PCR amplified by using primers for unidirectional sequencing by using fusion method (Ion Torrent Personal Genome Machine®, Life Technologies™). The PCR products obtained (with separate barcodes for each round) were resolved in a 3% agarose-TAE gel, the bands were excised, and the DNA was extracted from the gel by using a gel extraction kit (QIAquick Gel Extraction Kit, Qiagen). Next, equimolar amounts of DNA amplicon from each rounds were pooled and sent to sequence on a Personal Genome Machine (PGM) System at the Duke Sequencing and Genomic Technology Shared Resource. Library pool quality control was performed using Qubit/Bioanalyzer. Emulsion PCR was performed on a Life Technology OneTouch 2 instrument with the Ion PGM Template OT2 200 Kit (cat# 4480974) following the manufacturer guidelines and recommendations. Enriched templated beads were loaded on a 318 v2 chip for sequencing with the Ion PGM Sequencing 200 Kit v2 (cat# 4482006).

For the HTS data analysis, reads were first filtered to include only those that were at least 70 nt in length and had the 3′ and 5′ adapter sequences with a 38–42 nt variable aptamer sequence in between. Aptamer sequences were collected and ordered in terms of abundance. Cluster centroids were defined as sequences that have the highest total read count across all samples and are not within 4 mismatches of a previously defined cluster centroid. The remaining sequences are then placed into clusters based on their distance from the centroid (defined by the number of mismatches between that sequence and the centroid). Reads that are equal distance between two centroids are considered “ambiguous.”

### 2.3. Northern Blot

Cells (0.5 × 10^6^ cells/well) were plated in 12-well plates the day before the experiment, as described above. 400 pM of the aptamers or the control library was incubated with cells in 600 *μ*L of DMEM + 10% FBS media for 1 hour at 37°C (tissue culture incubator) under the cell growing conditions. After the incubation, the cells were washed 3x in DPBS buffer containing magnesium and calcium chloride and treated with trypsin to dissociate them from the plates. The suspended cells were washed, and total RNA was isolated using TRIzol reagent. Next, total RNA (3 *μ*g/lane) was resolved in a 15% urea-polyacrylamide gel (Bio-Rad) in 0.5x TBE buffer. The resolved RNA was transferred to a nylon membrane (GeneScreen Plus™ hybridization transfer membrane) in 0.5x TBE buffer at 20 volt (4°C for 4 hours). After the transfer, the nylon membrane was UV-cross-linked (UV Stratalinker® 2400, Stratagene) and dried overnight at room temperature. The membrane was incubated with 5 mL of hybridization buffer (Perfect Hyb™ Plus, Sigma) for 2 hours at 40°C for blocking and subsequently incubated with 5′-[*γ*-^32^P]-radiolabeled probe BW28 (5′-TCGGGCGAGTCGTCTG-3′) overnight at 40°C. The membrane was washed 3 times at 40°C in 0.5x SSC buffer containing 0.1% SDS and quantified using the Strom 825 Phosphorimager (GE Healthcare). For loading control, the labeled membrane was stripped and reprobed with radiolabeled U6 small nucleolar (snoRNA) specific probe 5′-CACGAATTTGCGTGTCATCCTT-3′.

### 2.4. Cell Binding and Internalization Assay

The quantified bands were used to calculate the relative amounts of aptamer binding to cells. The ratio was calculated by dividing the band volumes of aptamer by the control U6 band:

(1) $$\begin{array}{c} \text{Ratio}=\frac{\left(\text{aptamer band}-\text{background}\right)}{\left(\text{U6 band}-\text{background}\right)}. \end{array}$$

The ratio thus obtained for each aptamer was then divided by the ratio obtained for the Sel 3 library binding for calculating relative enrichment:

(2) $$\begin{array}{c} \text{Relative enrichment}=\frac{\text{Aptamer ratio}}{\text{Sel 3 library ratio}}. \end{array}$$

Cells were incubated with RS after trypsin treatment for the assessment of aptamer internalization. To obtain the relative quantity of MiaPaCa-2 cell bound aptamers that were internalized, the ratios of the aptamer and U6 bands from the RS (+) treated cells were divided by the ratio obtained from the cells that were RS (−) untreated.

## 3. Results and Discussions

Towards this goal, we performed cell-SELEX against the human pancreatic cancer cell lines, MiaPaCa-2 (ATCC). We incorporated an additional selection pressure in the SELEX protocol by using a cocktail of RNAse (RiboShredder, RS) that was first described by Magalhães et al. [11]. Aptamers that bind to cells are often internalized. They bind to proteins on the cell surface that recycle between the cell membrane and the cell-interior. Thus, aptamers are transported into cells by the “piggy-back” transport mechanism [4]. The rationale behind using the RNAse cocktail was to select for aptamers that were bound and internalized by the cells. The aptamers that are internalized will be protected from the RS and would be propagated for selection, whereas unbound aptamers, or aptamers that only bind to the cell surface but are not internalized, will be cleaved by the action of RS [11].

**Figure 1:**
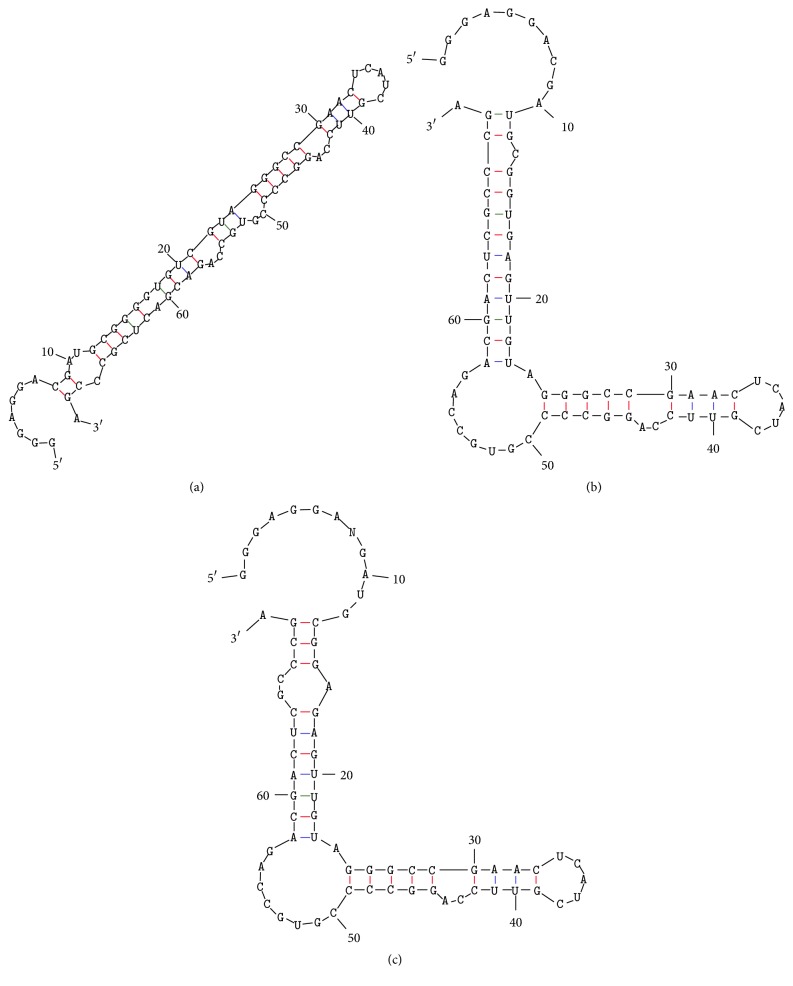
Secondary structure of the identified clones (a) M7-14, (b) P7-1, and (c) P7-14 predicted by the M-fold program [7].

After seven rounds of cell-SELEX, the selected pools of RNAs (round 1 through 7) from the MiaPaCa2 selection were subjected to high throughput sequencing (HTS) [13] in order to identify aptamers that were enriched during selection. Two major sequences were obtained that demonstrated progressive enrichment over rounds (Table 1(a); Supplementary File S2, in Supplementary Material available online at https://doi.org/10.1155/2017/4943072). In order to verify the HTS data we also cloned the last round of SELEX (round 7) and performed Sanger sequencing. The same sequences that were identified by HTS were identified by Sanger sequencing (Table 1(b), Figure 1).

**Figure 2:**
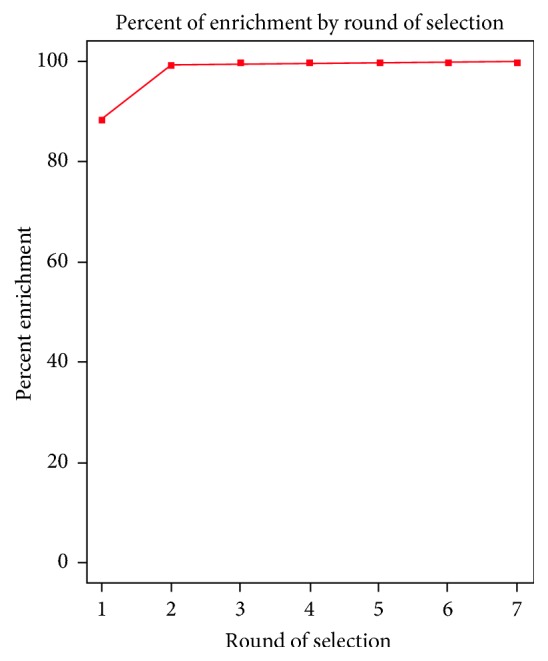
Percentage of enrichment of rounds (round 1 through 7) of the sequences obtained from MiaPaCa-2 HTS analysis; % Enrichment = 1 − (Unique sequence in individual round/Total sequence in individual round) × 100 [8].

Next, we performed bioinformatics analysis of the HTS data to monitor the progress of selection by following the percentage enrichment of the SELEX rounds (Figure 2). Interestingly, the winning sequences were highly enriched very early during the cell-SELEX. Even after performing a single round of cell-selection there was an enrichment of more than 88%. By the second round, the percentage of enrichment reached a value of greater than 99%, and there was very little enrichment in subsequent rounds. This early enrichment might be the consequence of proteins or other macromolecules that are present on the cell surface of pancreatic cancer cells that have high affinity for these aptamer sequences (Table 1(a)). Additionally, the absence of nonspecific blocking agents such as the tRNA or salmon sperm DNA during cell-SELEX might have accentuated the problem of “stickiness” of these cell surface residues to these aptamer sequences, resulting in their very early selection.

Additionally, to ascertain that the early selection of the aptamer is not a function of contamination, a control experiment was performed, where a single round of “mock” SELEX was done by using similar reaction conditions as mentioned for the cell-SELEX, but without cells. No detectable RNA was recovered after the reverse-transcription and PCR reactions, indicating that the early selection of the RNA aptamer is not a consequence of contamination (data not shown).

We also performed an independent SELEX using another pancreatic cancer cell line, Panc-1 (ATCC). Using the same protocol as described for the MiaPaCa-2, seven rounds of cell-SELEX were performed and the seventh round was cloned and sequenced. Interestingly, we obtained the same aptamer species, albeit with different percentages of representation, as were present in the seventh round on MiaPaCa-2 selection (Table 1(c)). These data support the hypothesis that certain species of nucleic acid aptamers are preferentially selected due to the presence of high affinity or high abundant cell surface binders present on the pancreatic cancer cells. Notably, the aptamer sequence for M7-14 comprised more than 63% of the sequences that were present in round 1 (Table 1(a)).

It should be noted that though in theory the identification of common sequence(s) in two independent selections with an *N*_40_ (variable region of the Library) is very low, we reason that when the starting DNA template is transcribed, specific sequences may be represented multiple times in the starting RNA library, which is then used for separate selections. Therefore, the presence of the same sequence(s) in independent selections is not necessarily the result of cross-contamination.

Due to the unexpectedly high enrichment of this sequence(s) in the early rounds of both selections, the initial Sel 3 (library) was cloned, and 11 “random” clones were sequenced. Sequence alignments of these clones confirmed the randomness of the variable region (Supplementary Figure S1), indicating that the early enrichment of this aptamer sequence was not a consequence of bias in the initial starting library.

The RiboShredder (RS) (RNase used in the cell-SELEX protocol) is a cocktail of proprietary, optimized blend of endo- and exo-RNase that completely degrades both modified and unmodified RNA. In previous studies, we have consistently observed that all the 2′-fluoro-pyrimidine-modified RNA aptamers that we tested are completely degraded when treated with this enzyme. Similar observations have been made by independent laboratories with other 2′-fluoro-modified RNA aptamers [11]. To further test this observation, we subjected radiolabeled, 5′-[*γ*^32^-P]-M7-14 aptamer to RS treatment (using similar conditions used during the cell-SELEX). No detectable bands were observed (compared to the untreated 5′-[*γ*^32^-P]-M7-14 aptamer) when resolved in a 12% polyacrylamide-urea gel and autoradiography was performed (data not shown), indicating that the aptamer is not inherently nuclease resistant.

Next, in order to study the cell binding properties of the selected aptamers, we performed flow cytometric (FCM) analysis by using fluorescently labeled aptamers [14]. We observed very modest binding (Supplementary Figure S2). When we performed similar analysis with different rounds of selection, we observed no increase in binding (data not shown). The method of end-labeling with biotin and streptavidin-conjugated fluorescent dye might induce oligomerization of the aptamers that could interfere with cell binding and internalization. Another possibility—not mutually exclusive with the former–is that the fluorescent tagging method used here affects the secondary structures of the selected aptamers.

**Figure 3:**
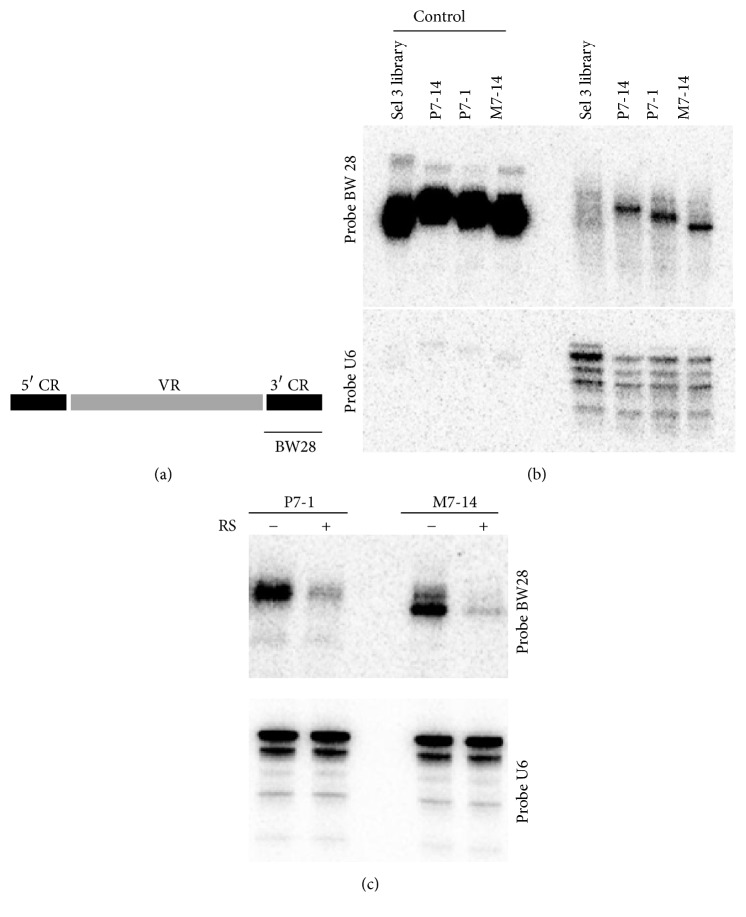
Northern blot analysis of the aptamer clones: (a) 5′-[*γ*-^32^P]-radiolabeled probe BW28, complementary to the 3′ constant region (CR) of the aptamer library, was used for the Northern blot analysis. Variable region (VR). (b) Total RNA (3 *μ*g/lane), isolated from the MiaPaCa-2 cells after incubating them with the aptamers, were subjected to Northern blot analysis using the 5′-[*γ*-^32^P]-BW28 (upper panel). For loading control, the nylon membrane was reprobed with the 5′-[*γ*-^32^P]-U6 snoRNA probe (lower panel). Purified aptamers (0.5 *μ*g/lane) and the starting library (Sel 3) were used as loading controls. Compared to the control (Sel 3 library), P7-14, P7-1, and M7-14 displayed 20, 17, and 24 times more binding to the MiaPaCa-2 cells, respectively. (c) To quantify the percentage of the aptamers internalized in the cells, the total RNA isolated form the MiaPaCa-2 cells after incubating them with the aptamers (P7-1 and M7-14) and with (+) or without (−) the RiboShredder (RS) treatment was subjected to the same Northern blot analysis as described above. 10.5% and 27% of the bound M7-14 and P7-1 aptamers were protected from the RS treatment, respectively, due to their internalization in the MiaPaCa-2 cells.

In order to assess cell binding and internalization of the selected aptamers without the need for labeling, we performed Northern blot analysis. MiaPaCa-2 cells were incubated with the aptamers, and total RNA isolated from the cells were subjected to Northern blot analysis by using a 5′-[*γ*-^32^P]-radiolabeled probe (BW28) that was complementary to the 3′ constant region of the aptamer library (Figure 3(a)). All of the selected aptamers demonstrated enrichment for binding to cells, as compared to the control library (Figure 3(b)). Similar data were obtained when Panc-1 and HPDE [15] (human pancreatic ductal epithelial) cells (normal pancreatic cells) were used for the assay (data not shown).

When we performed the same assay in conjunction with RS treatment, we observed that a fraction of the selected aptamers were internalized by the cells (Figure 3(c)). It should be noted that although all the aptamers were of the same size (70 nucleotides), they had slightly different migration pattern in the denaturing urea-polyacrylamide gel (Figures 3(b) and 3(c)). Highly stable structures, such as aptamers that are not completely denatured in the urea-polyacrylamide gel, can be the cause of this aberrant migration pattern. It should be noted that the selected aptamers have high G (guanosine) content that can induce highly structured G-quartet formation.

**Figure 4:**
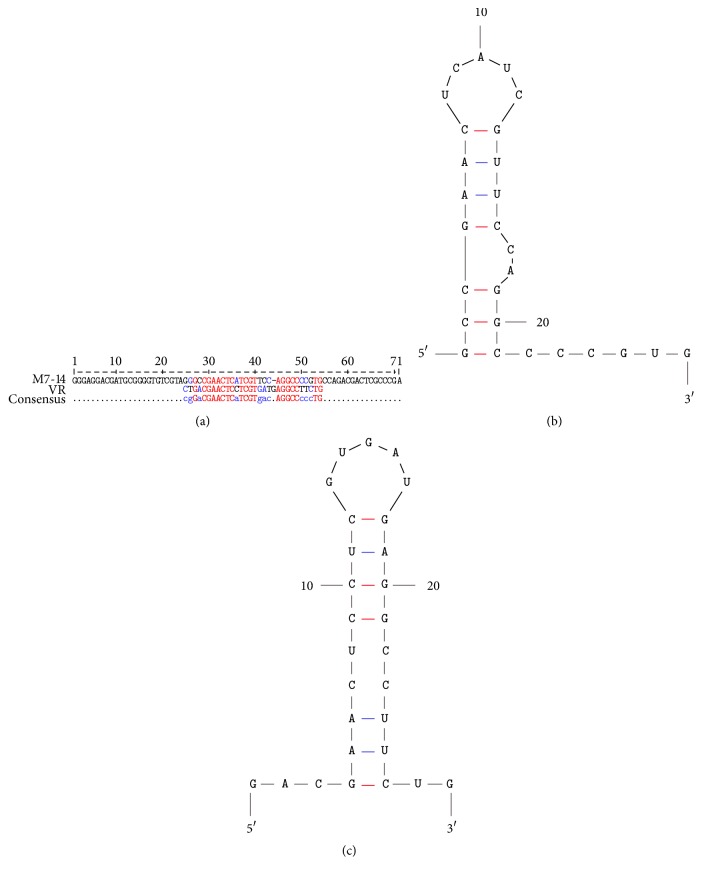
The sequence alignments of M7-14 and the variable region (VR) of the C3 aptamer [5]. The sequences were analyzed by using Multalign [9] program; 64% of the nucleotides were identical between the C3 VR and the M7-14 VR. The nucleotides that are identical between the two sequences are denoted in red font. The secondary structure of the aligned sequences for (b) M7-14 and (c) C3 VR, as predicted by the M-fold [7], identifies a common structural motif.

Interestingly, in a previously published report by Gourronc et al., the authors performed a positive-negative cell-SELEX strategy with the intention to select for aptamers that are specific for human papillomavirus type 16 (HPV16) E6/E7 transformed tonsillar epithelial cells [5]. These authors performed several rounds of stringent positive-negative selection by using HPV16 E6/E7 transformed tonsillar epithelial cells as positive and the corresponding nontransformed, human tonsillar epithelial cells (HTEC) as negative SELEX targets. When we analyzed the sequence of our aptamer (M7-14) against one of the predominant aptamers (C3) selected by this group, we observed that the variable region of C3 and part of M7-14's variable region were 64% identical (Figure 4(a)). It is extremely unlikely that aptamers against two different targets generated from two different libraries with a 40-nt random region (4^40^ different sequences) could be more than 50% identical by chance alone.

Notably, the secondary structure of the C3 variable region and the corresponding M7-14 variable region (predicted by the M-fold program [7]) had very similar structural motifs (Figures 4(b) and 4(c)). Interestingly, these variable regions have domain-like characteristics, in the sense that they retain their same secondary structure even when the rest of the aptamer sequence is absent (Figures 1(a), 4(b), and 4(c)).

We chemically synthesized (solid-phase phosphoramidite synthesis) part of the VR M7-14 region (21 nucleotides) that retained the same secondary structure as the full-length aptamer (predicted by the M-fold program) with 5′ Alexa488 fluorescent dye and performed flow cytometric analysis using Panc-1 cells. We observed that the VR M7-14 region confirms its cell binding nature and indicates that the “sticky” property can be attributed to this “domain” structure (Supplementary Figure S3).

Obtaining similar sequences from two independent selections and from selections performed by separate groups, where both the target cells and the aptamer libraries were different, strongly suggests that there is a strong selection pressure to select these sequences over the vast number of other sequences that are present in the aptamer library. It is unclear whether these sequences are binding to specific cell surface targets or whether these sequences mediate internalization by some other mechanism.

Additionally, these authors also refer to the fact that—despite performing positive-negative selections and using blocking agents such as tRNA to minimize nonspecific binding—most of the selected aptamers demonstrated only modest specificity towards the HPV16 transformed cells. C3 showed very modest preference for binding to the HPV-16 transformed cells over the control nontransformed cells that were used for the negative selection. These authors also mention their difficulty with flow cytometry, namely, the modest shift when fluorescently labeled aptamers were used for cell binding assays. It is possible that certain aptamer sequences or structures are not amenable to the 3′ end biotin-streptavidin-fluorophore tagging method that we often employ for cell binding analysis.

Taken together, these data suggest that we have selected a dominant aptamer sequence that either binds with low affinity to a highly abundant cell surface macromolecule or binds with very high affinity to a less abundant surface target. These highly abundant or high affinity targets are responsible for the generation of aptamers that take over the selection very early during SELEX, and—once selected—these aptamer species are propagated at the expense of other sequences (Figure 2, Table 1). These macromolecules seem to be common cell surface proteins, and, as a consequence, the aptamers selected against them do not show the specificity that is desired out of cell-SELEX.

We hypothesize that—by using the identified sequence as a blocking agent for these interactions—we can mask these common targets and thus allow for the selection of targets that are relatively rare. An alternative approach could be to deselect (remove) these aptamer sequences during selection rounds by using complementary oligonucleotides to block their amplification. It should be noted that this approach has successfully been used previously in a selection against a complex proteome (plasma) where the dominant aptamer sequence that bound to the most abundant protein (prothrombin) was deselected to redirect the SELEX against less abundant targets [16]. Hopefully, these approaches would improve upon the specificity of cell-SELEX strategies.

## 4. Conclusions

We performed cell-SELEX with the goal of identifying aptamers that specifically bind to pancreatic cancer cells; an additional selection pressure was applied by using RNAse to remove surface-binding aptamers and select for cell-internalizing aptamers. A common RNA sequence was identified from independent cell-SELEX procedures against two different pancreatic cancer cell lines, indicating a strong selection pressure towards this sequence from the large pool of other available sequences present in the aptamer library. Since binding/internalization of the aptamer could only reliably be detected by Northern blot, we did not screen many other cell lines for binding. It is conceivable that an aptamer could be “pancreas-specific,” even if not specific for the pancreatic cancer cell lines. However, the identification of a similar sequence motif in previously published aptamers against unrelated cell lines leads us to believe that the identified sequence forms a structural motif that binds to a common surface protein, which either is highly abundant or has strong affinity for the selected aptamer sequence. Deselecting (removing) this sequence during cell-SELEX may increase the probability of identifying aptamers against cell type-specific targets on the cell surface.

## Supplementary Material

Supplementary Table: High throughput sequences (HTS) Round R1 through R7: The sequences obtained from the HTS (R1 through R7), their respective counts and the percentage of their representation in each round are tabulated.
Supplementary Figure 1: Sequence alignment of M7-14 (aptamer) and Sel 3 (starting library) clones: Sequence alignment of Sel 3 clones confirmed the randomness of the variable region, indicating that the early enrichment of M7-14 sequence was not a consequence of bias in the initial starting library.
Supplementary Figure 2: Cell internalization assay of the selected aptamers by flow-cytometry: SA-PE labeled aptamers and the control Sel 3 library (180 nM) were incubated with the Panc-1 cells for 30 minutes at 37^⁰^C.
Supplementary Figure 3: Cell internalization assay of the Variable Region (VR M7-14, 21 nt “domain”) by flow cytometry.

## Figures and Tables

**Table 1a:** (a) Major sequences obtained from the high throughput sequencing (HTS) analysis of MiaPaCa2 (R1 to R7) cell-SELEX

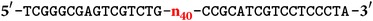	Clone	R1%	R2%	R3%	R4%	R5%	R6%	R7%
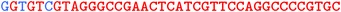	M7-14	63.5	79.8	77.0	68.0	79.4	82.9	78.8
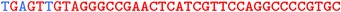	P7-1	6.03	7.90	10.2	13.8	7.22	4.38	7.60
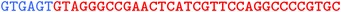	P7-1.1	1.00	0.70	1.18	3.91	1.89	2.79	2.80

**Table 1b:** (b) Sequences of clones from MiaPaCa-2 (R7) cell-SELEX

Clone	% of pool	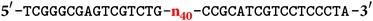
M7-14	86	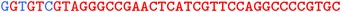
P7-1	13	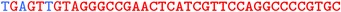

**Table 1c:** (c) Sequences of clones from Panc-1 (R7) cell-SELEX

Clone	% of pool	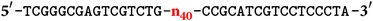
M7-14	45	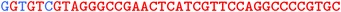
P7-1	45	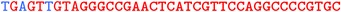
P7-14	9	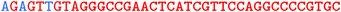

(a) HTS data analysis of sequences (rounds R1 through R7) obtained from the MiaPaCa-2 cell-SELEX identified three major sequences designated as M7-14, P7-1, and P7-1.1. The percentages of sequences identified in each rounds are tabulated. The variable region of the aptamers are represented in red, the flanking constant regions of the aptamers are in black, and the nucleotide residues that varied between all clones “mutations” are represented in blue. All the “mutations” were restricted outside the motif sequence region (red bold font), emphasizing the selection pressure to conserve the motif sequence and the structure. The aptamer cluster for all the sequences that were obtained from the HTS is presented in the Supplementary File (S2). (b) Sequences of the clones identified from round 7 (R7) of the MiaPaCa-2 cell-SELEX and their percentage of representations in the R7 sequence pool. (c) Sequences of the clones identified from round 7 (R7) of the Panc-1 cell-SELEX and their percentage of representations in the R7 sequence pool.
