# Supplementary material for: Cell-SELEX Identifies a “Sticky” RNA Aptamer Sequence
Source: J Nucleic Acids. 2017 Jan 17;2017:4943072. doi: 10.1155/2017/4943072 (PMC5282457; doi:10.1155/2017/4943072)
Supplement: Supplementary file 2 [file 4943072.f2.docx]

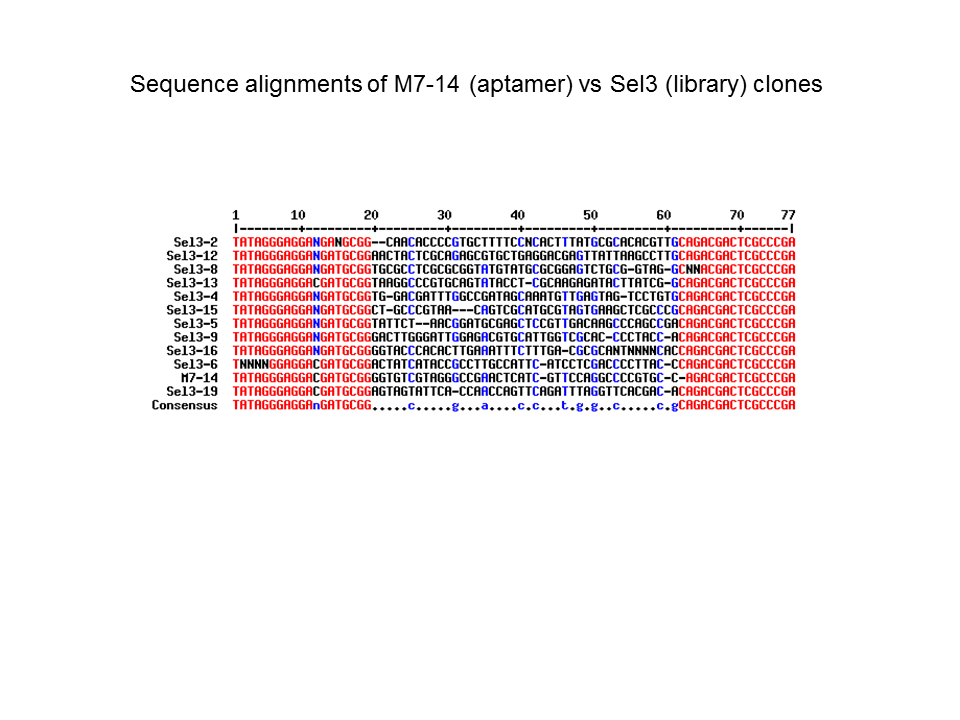


**Supplementary Figure 1:** **Sequence alignment of M7-14 (aptamer) and Sel 3 (starting library) clones:** Sequence alignment of Sel 3 clones confirmed the randomness of the variable region, indicating that the early enrichment of M7-14 sequence was not a consequence of bias in the initial starting library.


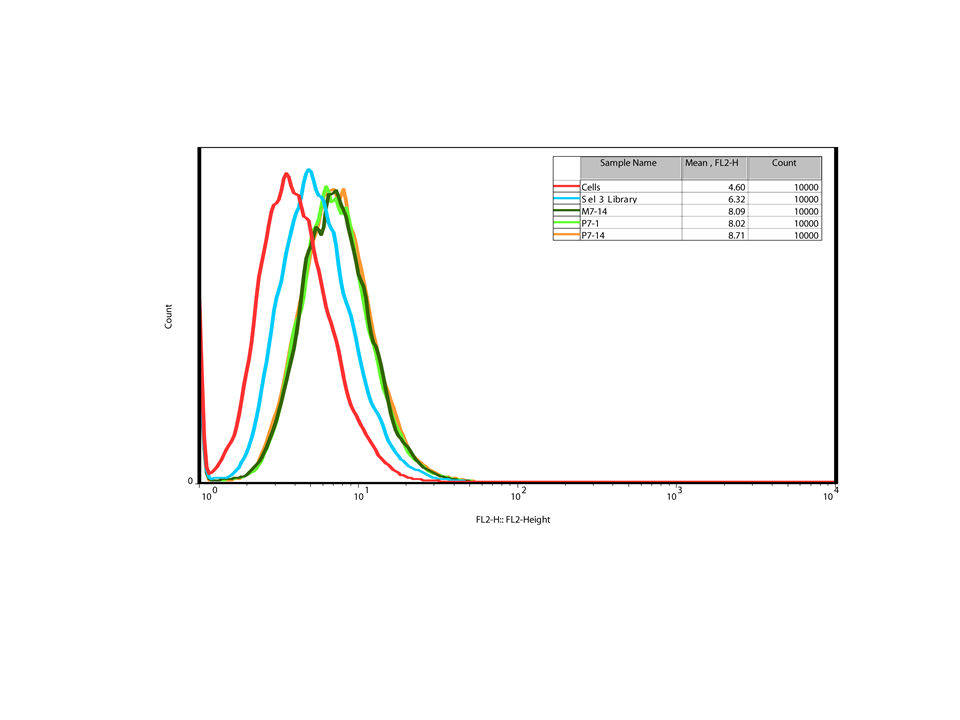


**Supplementary Figure 2: Cell internalization assay of the selected aptamers by flow-cytometry:** SA-PE labeled aptamers and the control Sel 3 library (180 nM) were incubated with the Panc-1 cells for 30 minutes at 37⁰C. Following which, the cells were treated with 2µl of Riboshredder at 37⁰C for 5 minutes. The cells were then washed in DPBS buffer containing MgCl_2_ and CaCl_2_ and then treated with trypsin to remove the cell surface proteins associated with the labeled aptamers. The pelleted cells were then washed in DPBS buffer containing MgCl_2_ and CaCl_2_ and analyzed by flow cytometry.


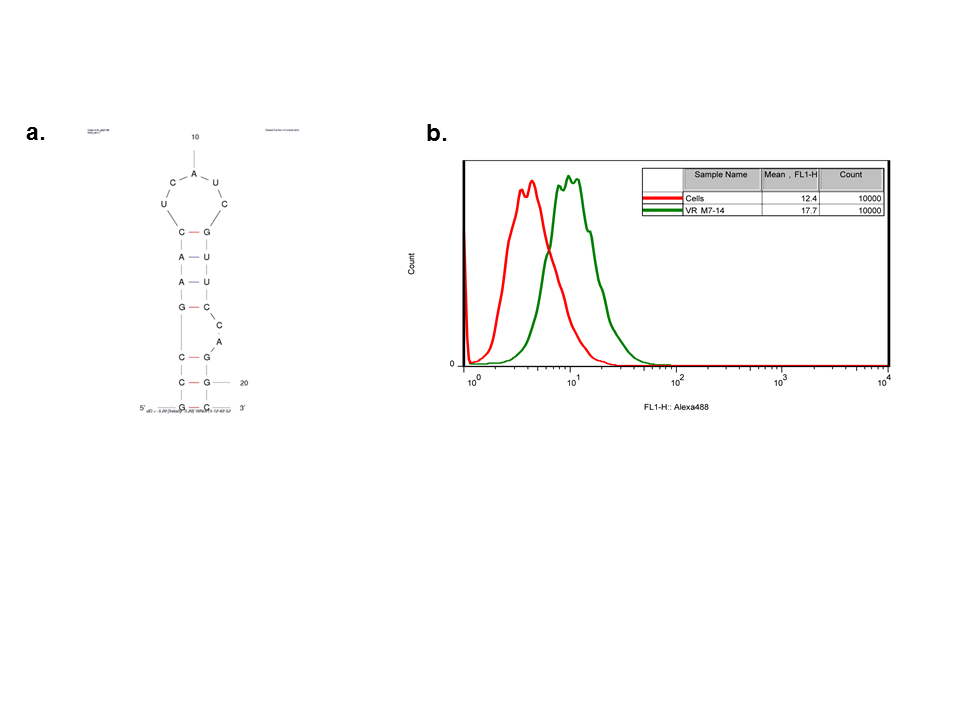


**Supplementary Figure 3: Cell internalization assay of the Variable Region (VR M7-14, 21 nt “domain”) by flow cytometry:** a) M-fold prediction of the secondary structure of the 21 nucleotides VR “domain”. b) VR M7-14 (21 nt “domain”) conjugated with 5ꞌAlexa 488 dye was incubated with Panc-1 cells (200 nM) for 1 hour at 37⁰C. Following incubation, the cells were washed in DPBS buffer containing MgCl_2_ and CaCl_2_ and then trypsinized to remove the cell-surface adhering aptamers. The pelleted cells were then washed in DPBS buffer containing MgCl_2_ and CaCl_2_ and analyzed by flow cytometry.
